# Respiratory dysfunction in two rodent models of chronic epilepsy and acute seizures and its link with the brainstem serotonin system

**DOI:** 10.1038/s41598-022-14153-6

**Published:** 2022-06-17

**Authors:** Hayet Kouchi, Michaël Ogier, Gabriel Dieuset, Anne Morales, Béatrice Georges, Jean-Louis Rouanet, Benoît Martin, Philippe Ryvlin, Sylvain Rheims, Laurent Bezin

**Affiliations:** 1 CRNL, Lyon Neuroscience Research Center, Inserm U1028, CNRS UMR5292, Lyon 1 University, 95 bd Pinel, 69500 Bron, France; 2 Epilepsy Institute IDEE, Bron, France; 3 IRBA, Institut de Recherche Biomédicale des Armées, Brétigny-sur-Orge, France; 4 Univ Rennes, CHU Rennes, Inserm, LTSI (Laboratoire de Traitement du Signal et de L‘Image), UMR-1099, Rennes, France; 5 Laboratoire d’Ecologie des Hydrosystèmes Naturels et Anthropisés, U5023, Lyon 1 University, Lyon, France; 6 Department of Clinical Neurosciences, Centre Hospitalier Universitaire Vaudois, Université de Lausanne, Lausanne, Switzerland; 7 Department of Functional Neurology and Epileptology, Hospices Civils de Lyon, Lyon, France

**Keywords:** Metabolism, Respiration, Diseases of the nervous system, Epilepsy

## Abstract

Patients with drug-resistant epilepsy can experience respiratory alterations, notably during seizures. The mechanisms underlying long-term alterations in respiratory function remain unclear. As the brainstem 5-HT system is a prominent modulator of respiratory function, this study aimed at determining whether epilepsy is associated with alterations in both the respiratory function and brainstem serotonin (5-HT) system in rats. Epilepsy was triggered by pilocarpine-induced *status epilepticus* in rats. Our results showed that 30–50% of epileptic (EPI) rats exhibited a sharp decrease in oxygen consumption (SDOC), low metabolic rate of oxygen, and slow regular ventilation (EPI/SDOC + rats). These alterations were detected only in rats with chronic epilepsy, independent of behavioral seizures, were persistent over time, and not associated with death. In these rats, 5-HT fiber density in the nucleus *tractus solitarius* was lower than that in the control and EPI/SDOC− rats. Both EPI/SDOC + rats and DBA/2 mice that present with audiogenic-induced seizure followed by fatal respiratory arrest—a model of sudden and expected death in epilepsy—had increased transcript levels of tryptophan hydroxylase 2 and 5-HT presynaptic transporter. Thus, our data support that 5-HT alterations are associated with chronic and acute epilepsy-related respiratory dysfunction.

## Introduction

The alteration of respiratory function is a chronic condition in epileptic patients that may represent a significant health concern, particularly for those with drug-resistant epilepsy^1,2^. For example, 33–86% of seizures expressed by patients with drug-resistant epilepsy are subsequently associated with an oxygen desaturation of < 90%^3,4^. In addition, the prevalence of obstructive sleep apnea (OSA) syndrome is twofold higher in epileptic patients compared to the general population^5,6^. Finally, it may be that preexisting respiratory dysfunction, particularly during the interictal period, represents a risk factor for sudden unexpected death in epilepsy (SUDEP), which results primarily from postictal central apnea^3^. The first objective of our study was to characterize in a preclinical model of drug-resistant temporal lobe epilepsy (TLE) respiratory disorders that might exist during the interictal period. Epilepsy following pilocarpine-induced status epilepticus (Pilo-SE) in rats is one of the most studied models of TLE. Like patients with TLE, rats that became epileptic after Pilo-SE are notably characterized by chronic, spontaneous, and drug-resistant seizures^7–9^. Respiratory function of such rats has been studied previously after the onset of epilepsy and independent of seizure activity. However, this required placing the animals under anesthesia and mechanical ventilation, a condition that can affect respiratory outcomes and limit interpretations^10^. We therefore first explored in unanesthetized and unrestrained rats subjected to Pilo-SE whether respiratory alterations occurred during epileptogenesis or after the onset of epilepsy

It is also crucial to fill in the knowledge gap regarding epilepsy-related respiratory alterations and the underlying pathological mechanisms. In this regard, the serotonin (5-HT) system may play a key role, since it has been involved in both physiological and pathophysiological mechanisms of breathing and epilepsy^11,12^. In addition, post-mortem examination of the brainstem of patients who died from SUDEP showed changes in the ventrolateral medulla (VLM)^13^, a region containing different populations of respiratory neurons, including those of the pre-Bötzinger complex, a kernel generator of the inspiratory rhythm^14^. Specifically, SUDEP patients presented with an alteration of the brainstem 5-HT system with a notable decrease in both tryptophan hydroxylase 2 (TPH2; rate-limiting enzyme in 5-HT synthesis) and the 5-HT presynaptic transporter (SERT; a protein that uptakes 5-HT from the synaptic cleft back to the presynaptic neurons) within the medullary raphe nuclei and/or the VLM^13^. In preclinical studies, DBA/1 and DBA/2 mouse strains are frequently used as SUDEP models, because they exhibit fatal respiratory arrest (FRA) after induction of an audiogenic seizure (AGS). In these mice, brainstem 5-HT abnormalities have been reported^15–17^, and FRAs were prevented when mice were pre-treated with fluoxetine before AGS, a selective 5-HT reuptake inhibitor (SSRI)^18,19^. These mice prone to AGS-induced FRA present however some limitations to perform a complete characterization of respiratory dysfunction in chronic epilepsy and its potential associated 5-HT abnormalities, because they are models of induced seizures and do not develop spontaneous recurrent seizures. Thus, the second objective of our study was to investigate brainstem 5-HT system alterations related to epilepsy in Pilo-SE rats by measuring the number of 5-HT-positive cell bodies, the density of 5-HT-positive terminals, and TPH2 and SERT mRNA levels. Given the preventive effect of SSRIs on FRA in DBA/2 mice, we also investigated whether TPH2 and SERT mRNA levels could distinguish DBA/2 mice prone to FRA from those that are not.

## Results

### Development of behavioral spontaneous recurrent seizures (SRSs) in rats after Pilo-SE

Rats that did not develop Pilo-SE or died during SE were excluded from experiments and their numbers are reported in Supplementary Information S1. SRSs were defined as detailed in the Methods section. Briefly, out of the 82 rats subjected to Pilo-SE in experiments 1–3, 59 developed at least two consecutive SRSs by the end of the 2^nd^ week post-SE, and these were then defined as EPI rats.

According to the evaluation of two experimenters, the number of recorded seizures (stages 3–5 of severity according to Racine’s scale) per 12 h of daylight period was 1.1 ± 0.1 in EPI rats at 3–8 weeks after SE. In experiment 1, none of the Pilo-SE rats developed two consecutive handling-induced seizures (HIS) at 1 week post-SE, in agreement with previous studies.

### A subset of EPI rats presented with abnormal pattern of oxygen consumption

In experiment 1, the metabolic rate of oxygen (⩒O_2_) in 20 EPI rats and 8 controls was recorded for 24 h each week, 1–8 weeks after SE. A thorough screening of ⩒O_2_ was then performed for each rat.

#### Presence of sharp decreases in oxygen consumption (SDOCs)

**Figure 1:**
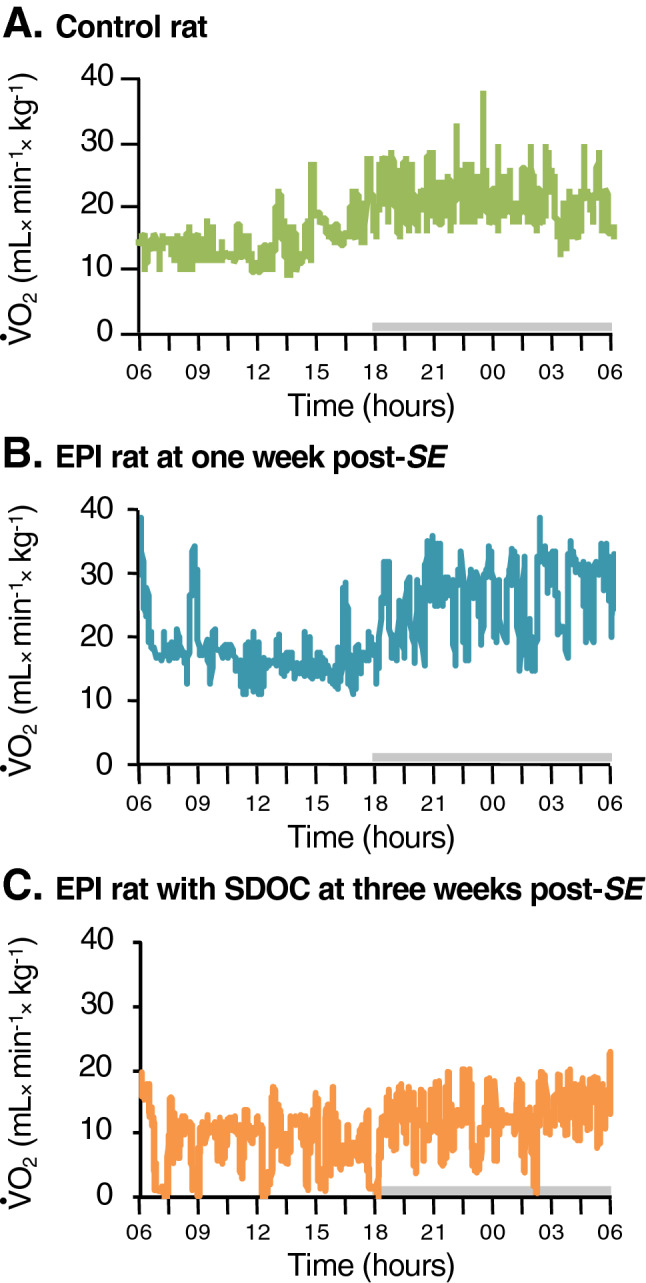
Detection of SDOC events during a 24-h monitoring of oxygen consumption in a subgroup of epileptic rats. Oxygen consumption (⩒O_2_) (light period from 06:00 am to 6:00 pm) was measured using respiratory thermochemistry. Grey bar above each x-axis indicates the dark period. A single profile was observed in control rats (**A**), while two distinct profiles were distinguished in epileptic rats (**B**,**C**). The first profile was similar to that observed in control rats (**B**), the second one was characterized by several episodes of sharp decrease in oxygen consumption (SDOC) (C), occasionally reaching zero. These SDOC events were only observed in epileptic rats, thereby dichotomizing two groups of epileptic rats (EPI/SDOC + and EPI/SDOC−).

**Figure 2:**
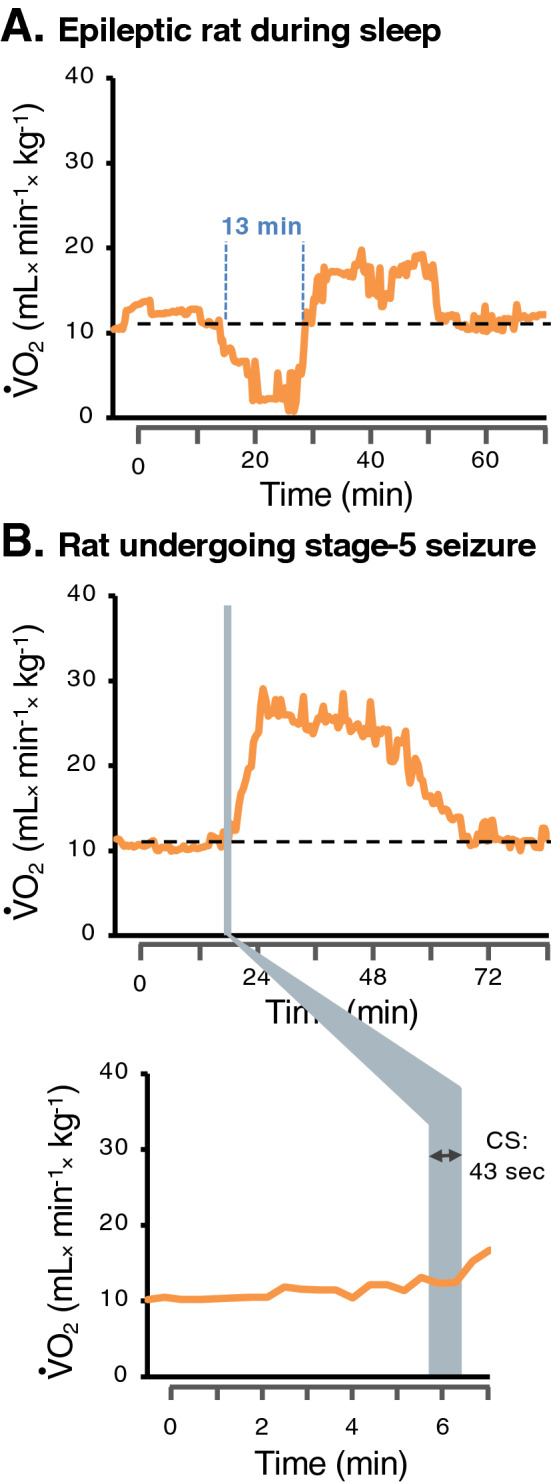
SDOC can occur independently of behavioral spontaneous seizures. (**A**) Example of an SDOC event recorded in an EPI rat at 5 weeks post-SE, when it was adopting a sleep posture. This SDOC event occurred during the light period and was identified with the decline in ⩒O_2_ values. No behavioral seizure was observed before, during, and after SDOC occurrence. In contrast, (**B**) a sharp increase in ⩒O_2_ was observed after a 43-s stage-5 seizure according to Racine’s scale in an EPI/SDOC + rat with ⩒O_2_ levels over the pre-ictal level for approximately 30 min.

Control rats (n = 8) presented with slight up and down fluctuations of ⩒O_2_ during both light and dark periods, as illustrated in one representative rat (Fig. 1A). For better characterization of daily ⩒O_2_ fluctuations, the average ⩒O_2_ for each rat during the light and dark periods was calculated. Subsequently, the lowest value below the average ⩒O_2_ was determined for each rat; this value ranged between 38 and 70% of their average ⩒O_2_, independently of the circadian rhythm. This pattern remained unchanged during recordings over the next 7 weeks. In rats subjected to Pilo-SE, the pattern of daily ⩒O_2_ was similar to that of controls during the first 2 weeks post-SE, as illustrated in one representative Pilo-SE rat (Fig. 1B). However, by the end of the 2nd week post-SE, two groups of EPI rats were identified according to ⩒O_2_ fluctuations. A subset of EPI rats presented with a large amplitude of ⩒O_2_ decrease (“Sharp Decrease(s) in Oxygen Consumption”; SDOCs), defined as a decrease greater than -62%, a percentage that corresponds to the maximal decrease of ⩒O_2_ in control rats. Such EPI rats were thus defined as EPI/SDOC + rats and their lowest ⩒O_2_ values ranged between 0 and 32% of the average ⩒O_2_, as illustrated at 3 weeks post-SE in one representative rat (Fig. 1C). In contrast, the remaining EPI rats (EPI/SDOC− rats) were similar to controls with regard to the lowest ⩒O_2_ values, which ranged between 41 and 67% of the average ⩒O_2_. We cannot exclude the fact that some SDOCs could be associated with the presence of focal seizures (with subclinical manifestation). However, video monitoring confirmed that SDOCs were not associated with behavioral seizures, as illustrated by an SDOC event that occurred while the rat was in a sleep-like position (Fig. 2A). In contrast, behavioral seizures were associated with a long-lasting increase in ⩒O_2_, as illustrated during a stage-5 seizure (Fig. 2B).

The highest ⩒O_2_ in control rats ranged from 164 to 229% of the average ⩒O_2_ (median = 183%). The highest fluctuations within the daily ⩒O_2_ pattern in EPI/SDOC + and EPI/SDOC− rats were not statistically different from controls, and ranged from 144 to 227% of the average ⩒O_2_ (median = 180%). However, greater increases in ⩒O_2_ were observed in EPI/SDOC + rats than in EPI/SDOC− rats (+ 93 ± 5% *vs* + 75 ± 4%; P = 0.01).

As a good indicator of the stability of the ⩒O_2_ pattern, the intra-individual variation (coefficient of variation, CV) of the daily ⩒O_2_ pattern (CV-⩒O_2_) was calculated independently of light and dark periods. The mean CV-⩒O_2_ of the control group was estimated to be 20.8 ± 0.5% and 22 ± 0.7% for the light and the dark periods, respectively. The mean CV-⩒O_2_ of EPI/SDOC− rats was almost similar to that of controls with 19.1 ± 0.8% and 22.8 ± 0.7% for the light and dark periods, respectively. However, the mean CV-⩒O_2_ was significantly higher in EPI/SDOC + rats, than in both the control (P = 0.002 and P = 0.004 for light and dark periods, respectively) and EPI/SDOC− groups (P < 0.0001 and P = 0.017 for light and dark periods, respectively), reaching 27.4 ± 2.9% and 26.5 ± 1.3% for light and dark periods, respectively.

#### *Low metabolic rate of oxygen in EPI/SDOC* + *rats*

Since ⩒O_2_ was measured weekly for all rats during 8 weeks, it was possible to retrospectively assign each EPI rat into either the EPI/SDOC + or the EPI/SDOC− group from the first week after SE.

**Figure 3:**
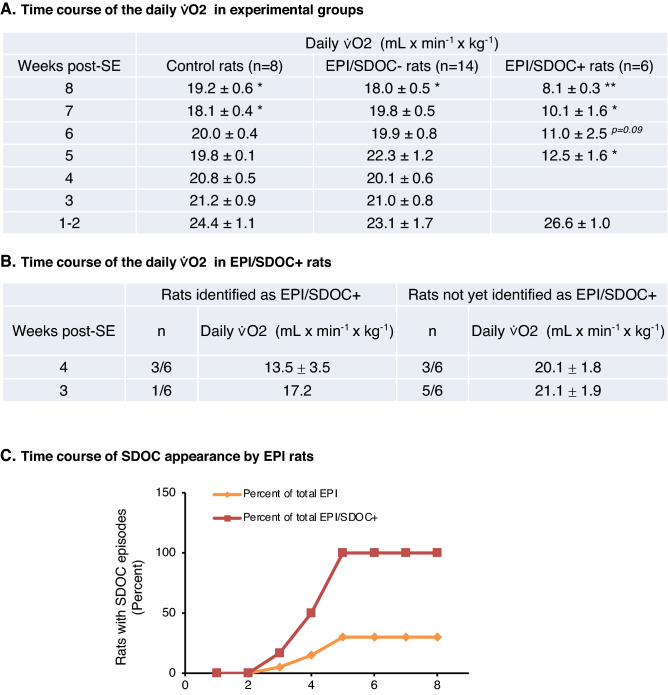
Time course of daily ⩒O_2_ pattern. Respiratory thermochemistry was recorded once a week for each rat, from the 1st to the 8th week post-SE. (**A**) Daily ⩒O_2_ was calculated for each group (control rats (n = 8), EPI/SDOC− rats (n = 14), EPI/SDOC + rats (n = 6)) from 1 to 8 weeks post-SE. (**B**) Daily ⩒O_2_ of animals was calculated at 3–4 weeks post-SE in animals identified as EPI/SDOC + rats or at 1–2 weeks post-SE in animals pending identification as EPI/SDOC + rats. Paired comparison of daily ⩒O_2_ over weeks showed statistical significance with only 1–2 weeks post-SE. Results are expressed as mean ± SEM of ⩒O2, *P < 0.05; **P = 0.01 in comparison to daily ⩒O_2_ at 1–2 weeks post-SE. (**C**) The first SDOC was detected in one rat (1/20) during the 3^rd^ post-SE week. The maximum number of EPI rats exhibiting SDOCs (EPI/SDOC + , 30%) was reached by the end of the 5th week post-SE. The proportion of EPI/SDOC + rats was expressed in percent of the total number of rats developing epilepsy (yellow curve) or in percent of the total number of rats presenting with SDOCs by the end of the 8th week post-SE (red curve).

We investigated the progression of daily ⩒O_2_ in each group. We first verified that SDOC events in EPI/SDOC + rats did not affect the average value of daily ⩒O_2_, both during the light (P = 0.86) and the dark (P = 0.93) periods. In order to compare the daily ⩒O_2_ during the 8 weeks of measurement between the three groups of rats, we used the data without SDOCs in EPI/SDOC + rats. We showed, in control and EPI/SDOC− rat groups, that daily ⩒O_2_ decreased progressively by 21.3 ± 2.4% (P = 0.05) and 22.0 ± 2.2% (P = 0.02), respectively, between weeks 1–2 and 8 post-SE. In contrast, the decrease observed in EPI/SDOC + rats was much larger during the same period (-69.5 ± 1.1%; P = 0.002), and reached -53 ± 6.0% (P = 0.02) by the fifth week post-SE (Fig. 3A). We also found that the decrease of daily ⩒O_2_ coincided with the onset of SDOC events (Fig. 3B).

In each group, we verified that daily ⩒O_2_ did not change significantly between 5 and 8 weeks post-SE; therefore, the average daily ⩒O_2_ has been calculated during that period for each group. We found that daily ⩒O_2_ was greatly reduced in EPI/SDOC + rats at 5–8 weeks post-SE compared to both EPI/SDOC− rats and controls (-39% ± 7% and -34% ± 7%, respectively). This decrease was observed during both the light period (EPI/SDOC + *vs.* EPI/SDOC− rats: P < 0.001; EPI/SDOC + *vs.* control rats: P = 0.002) and the dark period (EPI/SDOC + *vs.* EPI/SDOC− rats: P < 0.001; EPI/SDOC + *vs.* control rats: P = 0.002) (See supplementary Fig. S1A online). At weeks 1–2 post-SE, no difference was observed between groups during both the light and dark periods (See supplementary Fig. S1A online).

In the three groups of rats (controls, EPI/SDOC + and EPI/SDOC−), we evaluated the effect of the following three factors known to influence ⩒O_2_^20^: time (t_0_ corresponds to the time of induction of SE in EPI rats), body weight, and circadian rhythm. A full description of these effects is provided in Supplementary Information S1.

#### Characterization of SDOC

The number of SDOC events during the 24-h measurement ranged between 12 and 414 events (median = 44), with no difference between the dark and light periods. The median duration of SDOCs was 5 min (range: 20 s to 13 min). The longest SDOC event is illustrated in Fig. 2A; it lasted 13 min and occurred, according to the video, during the daylight period while the animal was in a sleep-like position without any evidence of behavioral seizure. Full description of the ⩒O_2_ pattern during seizures is provided in Supplementary Information S1.

Body position during SDOCs was different from the freezing posture frequently observed before rats have a behavioral seizure (stages 3 to 5 on Racine’s scale). Notably, rats always exhibited a quiet state during SDOC events. The proportion of EPI/SDOC + rats increased between weeks 3 and 5 post-SE and then stabilized (from weeks 5 to 8 post-SE). By the end of the 5^th^ week post-SE, 30% (6/20) of EPI rats exhibited SDOCs (Fig. 3C).

### Ventilatory and cardiac functions in epileptic rats

Experiment 2 aimed to further investigate the respiratory function by evaluating the ventilatory and cardiac functions of EPI rats during the chronic phase of epilepsy. It should be noted that we could not record respiratory variables using whole-body plethysmography before the 10^th^ week post-SE because rats were not able to remain quiet in the apparatus chamber, likely owing to enhanced reactivity to stressful situations.

#### EPI rats presented with a low steady respiratory pattern and enhanced ventilatory response to hypoxia and hypercapnia

Under normoxia, EPI rats (n = 16) presented a slow respiratory pattern with a significant decrease in minute ventilation (⩒_E_) compared to controls (n = 5) (− 12 ± 3%, P = 0.03) (See supplementary Fig. S2A online). The reduction of ⩒_E_ in EPI rats resulted from a decrease in respiratory frequency (f_R_) (− 12 ± 2% *vs.* controls, P = 0.001), while tidal volume (V_T_) was similar between EPI and control rats (P = 0.92). The decrease in f_R_ in EPI rats was related to a simultaneous increase in inspiratory time (T_I_) and expiratory time (T_E_), which reached + 20 ± 3% (P = 0.01) and + 10 ± 2% (P = 0.001) compared to the respective values measured in controls. Consequently, total respiratory cycle duration (T_tot_) was significantly higher in EPI rats than in controls (+ 14 ± 2%; P = 0.002) (See supplementary Fig. S2A online).

Regarding the stability of the breathing pattern, the coefficient of variation of breathing, apnea frequency, and duration were similar between EPI and control rats (P = 0.16, P = 0.61, and P = 0.09, respectively) (see supplementary Fig. S2B online).

Compared to control rats (n = 13), EPI rats (n = 18) showed greater hypoxia ventilatory response (HVR) with higher ⩒_E_ (+ 23 ± 6% *vs* controls, P = 0.007), higher f_R_ (+ 16 ± 2% *vs* controls, P < 0.001) and similar V_T_ (P = 0.06). Similarly, the hypercapnia ventilatory response (HCVR) was higher in EPI rats (n = 18) than in controls (n = 12) with greater ⩒_E_ (+ 14 ± 4% *vs* controls, P = 0.01) and greater f_R_ (+ 10 ± 3% *vs* controls, p = 0.02), while V_T_ remained similar to that of controls (P = 0.09).

### ***Abnormal ***⩒***O***_***2***_*** pattern is associated with pronounced decrease in ventilatory rhythm at rest***

Since oxygen is strictly provided during the ventilatory process, we aimed to compare ventilation between EPI rats with or without abnormal ⩒O2 patterns. In experiment 2, we were able to discriminate eight EPI/SDOC + rats, which presented with lower daily ⩒O_2_ compared to that of both EPI/SDOC− rats (n = 15, -51% ± 9%; P < 0.001) and control rats (n = 16, -41% ± 11%; P = 0.003).

Under normoxia, ventilation of EPI/SDOC− rats was significantly altered compared to that of control rats (Table 1A), but to a lesser extent than that of EPI/SDOC + rats. Indeed, compared to control rats, EPI/SDOC− rats presented a slight but not significant 6 ± 3% of reduction in ⩒_E_, associated with an 8 ± 2% of reduction (P = 0.015) in f_R_, a 13 ± 1% of increase (P < 0.001) in T_I_ and a 9 ± 2% of increase (P = 0.025) in T_tot_ (Table 1A). Furthermore, compared to EPI/SDOC− rats, EPI/SDOC + rats presented an additional 13 ± 3% of reduction (P = 0.013) in ⩒_E_, that resulted from an 8 ± 2% of reduction (P = 0.036) in f_R_, a 13 ± 3% of increase (P < 0.001) in T_I_ and an 8 ± 3% of increase (P = 0.013) in T_tot_ (Table 1A). Tidal volume (V_T_), the coefficient of variation of the breathing frequency, apnea frequency, and duration were similar between all groups (Table 1A). Under hypoxic and hypercapnic conditions, ⩒_E_, V_T_, and f_R_ were similar between EPI/SDOC + (n = 6) and EPI/SDOC− rats (n = 12) (Table 1B).

**Table 1:** Monitoring of cardiorespiratory functions in control and EPI rats between 12 and 15 weeks post-SE.

	Control (n=5)	EPI/SDOC- (n=8)	EPI/SDOC+ (n=8)	Ctrl vs SDOC- (P value)	Ctrl vs SDOC+ (P value)	SDOC+ vs SDOC- (P value)	
**(A) Basal respiratory variables under normoxia at 12 weeks post-*SE***							
**⩒**_**E**_ (mL min^−^1 × 100 g−1)	32 ± 1	30 ± 1	26 ± 1	*0.274 *	0.002	0.013	
fR (breath × min−1)	101 ± 3	93 ± 2	87 ± 2	0.015	<0.001	0.036	
VT (mL × 100 g−1)	0.32 ± 0.01	0.33 ± 0.01	0.31 ± 0.01	*NT *	* NT *	*NT*	
TI (ms)	207 ± 7	233 ± 3	263 ± 8	0.015	<0.001	<0.001	
TE (ms)	385 ± 12	413 ± 9	438 ± 10	*0.088 *	0.003	*0.083*	
Ttot (ms)	593 ± 14	646 ± 11	700 ± 17	0.025	<0.001	0.013	
Apnea frequency × min−1	0.32 ± 0.17	0.27 ± 0.13	0.10 ± 0.07	*NT *	*NT *	*NT*	
Apnea duration (ms)	1339 ± 67	1435 ± 65	1477 ± 75	*NT *	*NT *	*NT*	
Coefficient of variation	0.49 ± 0.01	0.53 ± 0.01	0.52 ± 0.02	*NT *	*NT *	*NT*	

% of baseline		Control (n = 13)	EPI/SDOC− (n = 12)	EPI/SDOC+ (n = 6)	Ctrl vs SDOC− (P value)	Ctrl vs SDOC+ (P value)	SDOC+ *vs *SDOC− (P value)
**(B) Respiratory response to hypoxia and hypercapnia at 13-14 weeks post-*SE***							
Hypoxia *9% of O2*	⩒_E_ (mL min−1 × 100g−1)	225 ± 9	273 ± 18	278 ± 19	0.019	0.046	*0.847*
	fR (breath × min−1)	146 ± 5	171 ± 4	165 ± 3	<0.001	0.024	*0.463*
	VT (mL × 100 g−1)	153 ± 5	169 ± 8	170 ± 7	*NT*	*NT*	*NT*
Hypercapnia *5% of CO2*	⩒_E_ (mL min−1 × 100g−1)	187 ± 6	211± 8	219 ± 14	0.028	0.023	*0.562*
	fR (breath × min−1)	128 ± 4	141 ± 4	141 ± 8	*NT*	*NT*	*NT*
	VT (mL × 100 g−1)	141± 3	148 ± 5	153 ± 6	*NT*	*NT*	*NT*

		Control (n = 3)	EPI/SDOC− (n = 3)	EPI/SDOC+ (n = 2)	Ctrl vs SDOC− (P value)	Ctrl vs SDOC+ (P value)	SDOC+ vs SDOC− (P value)
**(C) Cardiac frequency at 15 weeks post-*SE***							
Day	FC (beats × min−1)	315 ± 17	338 ± 11	333 ± 8	*NT*	*NT*	*NT*
Night	FC (beats × min−1)	332 ± 19	355 ± 9	351 ± 12	*NT*	*NT*	*NT*

		Control (n = 3)	EPI/SDOC− (n = 3)	EPI/SDOC+ (n = 2)	Ctrl vs SDOC− (P value)	Ctrl vs SDOC+ (P value)	SDOC+ vs SDOC− (P value)
**D- Body-temperature at 15 weeks post-*SE***							
Day	TB (°C)	37.5 ± 0.1	37.6 ± 0.1	37.7 ± 0.2	*NT*	*NT*	*NT*
Night	TB (°C)	38 ± 0.2	37.9 ± 0.1	38.1 ± 0.1	*NT*	*NT*	*NT*

Using plethysmography, ventilatory variables were measured under normoxic conditions (21% O_2_) at 12 weeks post-SE (A) and in response to hypoxia (9% O_2_) or hypercapnia (5% CO_2_) exposure between 13–14 weeks post-SE (B). Cardiac frequency (C) and body-temperature (D) were concomitantly evaluated at 15 weeks post-SE.

*Fc* cardiac frequency, *f*_*R*_ respiratory frequency, *T*_*B*_ body-temperature, *T*_*E*_ expiration time, *T*_*I*_ inspiration time, *T*_*tot*_ total respiratory cycle duration, *⩒*_*E*_ minute volume, *V*_*T*_ tidal volume. Statistics: *NT* not statistically significant.

#### No alteration in cardiac frequency and body temperature in EPI rats

The respiratory system, cardiac function, and body temperature are strongly related to each other. In this experiment, we showed that cardiac frequency and body temperature were similar between EPI/SDOC + rats (n = 2), EPI/SDOC− rats (n = 3), and control rats (n = 3) during both the light and dark periods (Table 1C,D).

### Brainstem 5-HT system of EPI rats

In experiment 3, 20 rats developed epilepsy. Among these rats, we identified eight EPI/SDOC + and eight EPI/SDOC− rats. Eight control rats were used in this study. The brainstem was processed for either histological (n = 3/8 rats in each group) or gene expression (n = 5/8 rats in each group) analyses.

#### Alterations in the density of brainstem 5-HT immunopositive processes in EPI rats

**Figure 4:**
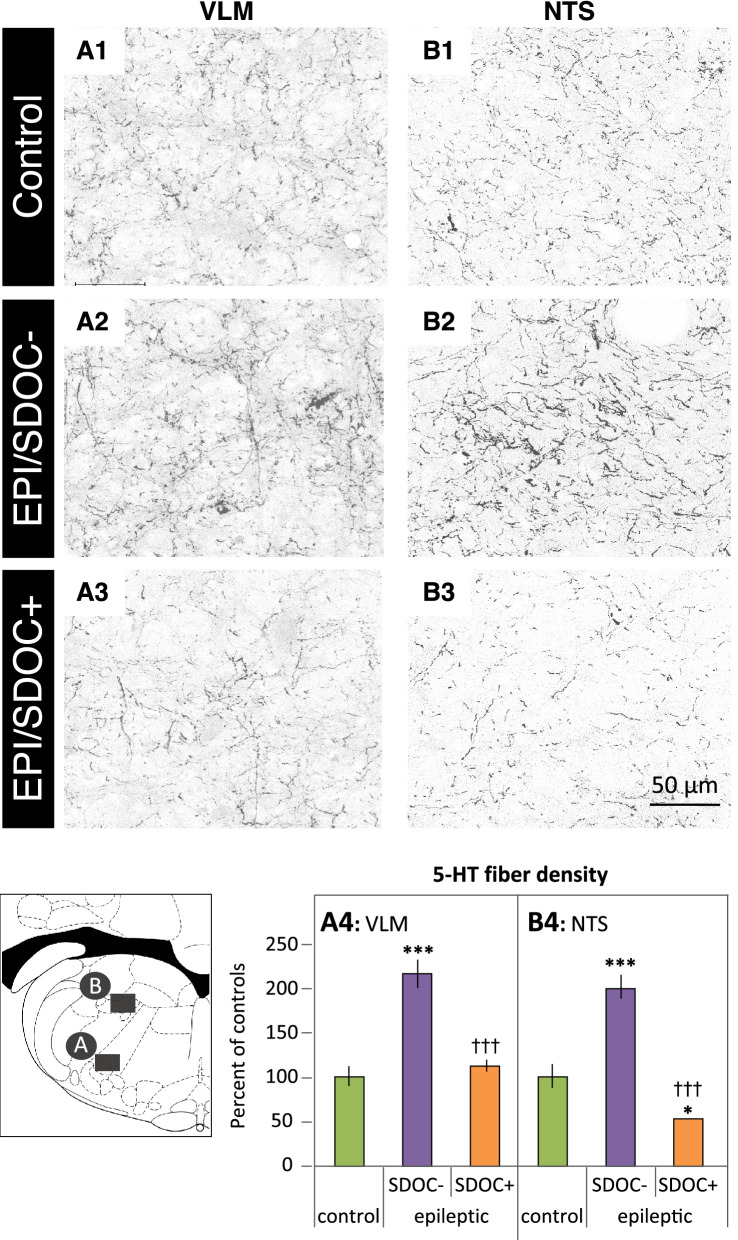
Semi-quantification of 5-HT immunopositive terminals in the caudal raphe nuclei within the medulla oblongata in control and EPI rats. Fluorescent 5-HT labeling was performed in sections selected at interaural − 2.60 mm within two brainstem regions involved in the regulation of respiratory rhythm generation: the ventrolateral medulla (VLM) and nucleus tractus solitarius (NTS). Images of 5-HT positive fibers were captured within the VLM (A1, A2, A3) and the NTS (B1, B2, B3) using confocal microscopy (× 63 objective). Density of 5-HT positive fibers was calculated in the VLM (A4) and the NTS (B4). Data are expressed as mean ± SEM (n = 3 per group). *P < 0.05; ***P < 0.001, compared to controls and †††P < 0.001, compared to EPI/SDOC−.

We determined whether metabolic and ventilatory alterations developed by EPI/SDOC + rats were associated with dysfunction of the brainstem 5-HT system. Therefore, we focused our investigation on caudal raphe nuclei, which are known to innervate neuronal groups closely implicated in the regulation of respiratory rhythm generation, such as the NTS and the VLM^14,21^. 5-HT-immunolabeling was detected in both processes and cell bodies. Within the caudal raphe nuclei (pallidus, magnus, and obscurus), although we observed a downward trend in epileptic rats, there was no statistical difference in the number of 5-HT-positive neuronal cell bodies between the three groups of rats (control rats: 22 ± 6, EPI/SDOC−: 18 ± 4, EPI/SDOC + : 13 ± 5, P = 0.339) (See supplementary Fig. S3 online). In addition, there was no difference in the surface area of 5-HT positive cell bodies (control rats: 383 ± 21 pixels^2^, EPI/SDOC− rats: 445 ± 37 pixels^2^, EPI/SDOC + rats: 397 ± 54 pixels^2^, P = 0.383), or in the index of 5-HT neuronal concentration (control rats: 109 ± 19 A.U., EPI/SDOC− rats: 125 ± 23 A.U., EPI/SDOC + rats: 119 ± 18 A.U., P = 0.864) between the three rat groups (See supplementary Fig. S3 online). The density of 5-HT immunopositive processes within both the VLM (Fig. 4A) and NTS (Fig. 4B) were significantly higher in EPI/SDOC− rats than in EPI/SDOC + rats and control rats (Fig. 4A4 and 4B4). Furthermore, the density of 5-HT-immunolabeled processes within the NTS was significantly lower in EPI/SDOC + rats compared to controls (Fig. 4B4).

#### *Alterations in brainstem transcript levels of TPH2 and SERT in EPI/SDOC* + *rats*

**Figure 5:**
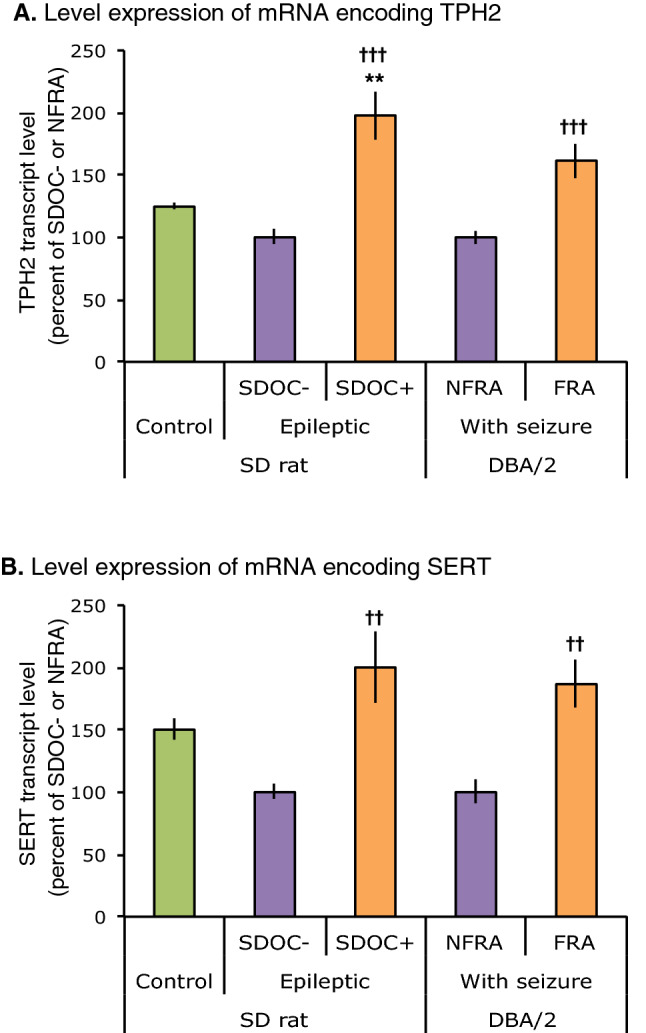
TPH2 and SERT mRNA transcript levels were measured in the brainstem of EPI rats and DBA/2 mice. Changes in brainstem levels of TPH2 (**A**) and SERT (**B**) transcripts were measured in EPI rats and DBA/2 mice at 8 weeks post-SE and 2–3 min after audiogenic seizure (AGS), respectively. In contrast to EPI rats, DBA/2 mice had no history of seizure before AGS. Some mice developed fatal respiratory arrest (DBA/2 with FRA) or non-fatal respiratory arrest (DBA/2 with NFRA) after AGS. TPH2 and SERT transcript levels in EPI/SDOC + rats and in DBA/2 mice with FRA are expressed as percentage (mean ± SEM) of EPI/SDOC− rats and DBA/2 mice with NFRA, respectively (n = 5 in each group). †P < 0.05, ††: P < 0.01, †††: P < 0.001, in comparison to EPI/SDOC− rats and DBA/2 mice with FRA. *P < 0.05; **P < 0.01, in comparison to control rats.

TPH2 transcript levels were significantly increased in EPI/SDOC + compared to both EPI/SDOC− rats and control rats. There was no statistical difference in the transcript levels of TPH2 between EPI/SDOC− and control rats (Fig. 5A). SERT transcript levels were significantly increased in EPI/SDOC + compared to EPI/SDOC− rats, but not when compared to control rats. In addition, SERT transcript levels did not differ statistically between control and EPI/SDOC− rats (Fig. 5B).

### Brainstem 5-HT system in DBA/2 mice prone to AGS

Previous studies demonstrated that almost 75% of DBA/2 mice developing AGS died from fatal respiratory arrest (FRA) following tonic hindlimb extension^22^. Among the remaining mice, 15% developed non-fatal respiratory arrest (NFRA), indicating that they spontaneously recovered from post-AGS respiratory arrest. The brainstem 5-HT system in DBA/2 mice exhibiting FRA in response to AGS was altered in comparison with C57Black/6 mice that did not exhibit AGS^16^. Thus, in experiment 4, we examined whether the difference in TPH2 and SERT gene expression that we observed between EPI/SDOC + rats and EPI/SDOC− rats was consistent in FRA and NFRA DBA/2 mice after AGS. Out of 11 male DBA/2 mice that underwent AGS with respiratory arrest, 64% presented with FRA (n = 7) and 36% with NFRA (n = 4). We found that the levels of TPH2 and SERT transcripts were significantly elevated in FRA DBA/2 mice compared to those in NFRA DBA/2 mice (Fig. 5A,B). Strikingly, the levels of TPH2 and SERT transcripts in FRA DBA/2 mice followed a trend similar to that of EPI/SDOC + rats when both were compared to their respective controls (NFRA DBA/2 mice and EPI/SDOC− rats, respectively).

## Discussion

In this study, we report chronic alterations in the respiratory system in epileptic (EPI) rats associated with 5-HT dysfunction within the brainstem.

### Exploration of respiratory function in epilepsy models

To date, studies in animal models of seizures, aimed at understanding respiratory dysfunction in the context of epilepsy, were mostly performed during the period surrounding seizures, i.e., during ictal and post-ictal periods^22–32^. Respiratory alterations were detected in these studies during short recording periods (2–30 min). In view of the respiratory disorders that can affect patients suffering from temporal lobe epilepsy^1,2,5,6^, our work, which was performed on a widely used preclinical model of this type of epilepsy (*status epilepticus* induced by intraperitoneal administration of pilocarpine, Pilo-SE rats), had two major objectives.

The first was to study the respiratory function in rats with a long history of epilepsy and at distance of brain remodeling period that follows the severe brain damage induced by Pilo-SE^33^. After a 10-week period of active epilepsy (12 weeks post-SE), we observed a decrease in breathing rhythm in epileptic (EPI) rats at rest. This result is different from that reported in a recent study showing no alteration in resting ventilation in rats 2–4 weeks after Pilo-SE^34^, probably due to the fact that we did not use the same strain of rats (Wistar vs. Sprague–Dawley in our study), or the same route of pilocarpine administration (intrahippocampally vs. intraperitoneally in our study). Nonetheless, we agree with the authors that the mechanisms leading to alterations in breathing may depend on the stage of epilepsy; in their study, the assessment was performed at an earlier stage compared to that in our study (chronic epilepsy stage).

The second objective was to perform respiratory function recording over a long duration (24 h) in order to extend the investigation beyond the period surrounding seizures. Using indirect respiratory thermochemistry, we performed a 24-h monitoring to determine the amount of oxygen consumed by the whole body. Subsequently, we provided evidence of the presence of abnormal ⩒O_2_ patterns in 30–50% of epileptic rats, which started with the installation of epilepsy (2–3 weeks post-SE). These EPI rats (EPI/SDOC + group) presented chronic ⩒O_2_ abnormalities characterized by low daily patterns, with intermittent and repetitive SDOC events.

Until now, both preclinical and clinical studies have investigated changes in the metabolic rate of O_2_ in epilepsy mostly at the brain level, reporting a decline in the activity of mitochondrial respiratory complexes in rats subjected to Pilo-SE and in patients with TLE^35,36^. In our study, we measured whole-body O_2_ intake, which is mostly related to gas exchange during the ventilatory process^37^. However, whole-body O_2_ intake can also be affected directly or indirectly by several factors, including age, circadian rhythm, body weight, body temperature, cardiac function, and activity^20,37–42^. None of these factors were found to be associated with abnormal pattern of ⩒O_2_ in our study. We then focused on ventilatory variables that may explain abnormal ⩒O_2_ patterns in a subset of EPI rats.

### Respiratory system at rest in a subset of EPI rats

The identification of heterogeneous phenotypes within a single disease is a common feature in human epilepsy, but has rarely been described in preclinical studies. One study dichotomized two groups of rats subjected to kainic acid-induced SE according to the extent of brain damage and brain metabolic alterations^43^. In our study, the EPI/SDOC + group presented with a decrease in daily ⩒O2 and a severe decrease in ⩒E compared to the EPI/SDOC− group.

During a 20-min monitoring period at rest, the decrease in both ⩒E and ⩒O_2_ has already been described as an inherent dysfunction in a genetic model of audiogenic seizure in the WAR rat strain^31^. Because of technical limitations (24-h ⩒O_2_ monitoring), we could not jointly assess ⩒E and ⩒O_2_ in our study, which prevented us from interrogating the potential direct relationship between ⩒E and ⩒O_2_. Nevertheless, we estimated the ⩒E/⩒O_2_ ratio for each rat, as an indicator of ventilatory efficiency; this ratio was significantly higher in EPI/SDOC + rats compared to EPI/SDOC− (+ 52%, P = 0.007) and healthy control rats (+ 35%, P = 0.04), and not statistically different between the latter rat groups.

High ⩒E/⩒O_2_ has been described as an index of poor gas exchange during physical exercise in individuals with heart failure disease^44^. Further exploration using blood oxygen measurements are required to determine the direct relationship between ⩒E and ⩒O_2_ in epileptic rats following Pilo-SE. Another limitation of this study was the short duration of the ventilatory recording compared with the 24 h of ⩒O_2_ assessment. A significant fluctuation of ⩒E could have possibly been observed, similar to those observed for daily ⩒O_2_, if the recording of ⩒E had been longer. Further studies with devices that can concomitantly measure both ⩒E and ⩒O_2_ over a long period of time in free-moving rats will help to establish a direct link between ⩒O_2_ and ⩒E profiles and more specifically during SDOC events.

Indeed, these events could represent a disruption in the O_2_ uptake balance. To our knowledge, events similar to SDOCs have never been reported, and could only be interpreted in light of our knowledge regarding dyspneic patterns. One possible explanation for SDOCs could be the presence of ventilatory pauses; however, they have to be extremely numerous and with a long duration to induce such a drop in ⩒O_2_. Another explanation could be a combination of several factors; for example, the lack of gasping, especially in hypoxic conditions, could lead to poor tissue oxygenation^45^. In EPI/SDOC + rats, ⩒E recording was too short to determine whether the absence of gasping could be part of their ventilatory dysfunction.

### Ventilatory regulation in EPI rats

We observed an increased HVR in EPI rats, in contrast to some studies that reported no alteration in HVR in Pilo-SE rats^10,34^. In addition, EPI/SDOC + and EPI/SDOC− rats exhibited sustained HCVR, whereas previous studies have reported a diminished response in Pilo-SE-induced epilepsy, rapid amygdala kindling model, and WAR rat model^31,34,46^. It is very difficult to compare our data with those in the literature owing to differences in the models used, timing of respiratory recordings, and severity of exposure to hypoxia and hypercapnia. Given that low HVR and HCVR are generally indicative of alterations in peripheral or central respiratory components, their maintenance in high levels in our study may indicate that if alterations occurred in early phases, they were compensated for, or repaired, in the late stages of epilepsy at which we performed our investigation.

### Brainstem 5-HT alterations

5-HT is mostly described as an excitatory drive towards the respiratory network, and subsequently the motor output^21^. Dysregulation of brainstem neuronal groups involved in the control of breathing, particularly those of the 5-HT system, has been found in patients with drug-resistant epilepsy and has been associated with an increased risk of SUDEP^13^. We found that brainstem 5-HT innervation was impaired in EPI rats compared to control rats, with notable differences between EPI/SDOC + and EPI/SDOC− rats. Indeed, EPI/SDOC− and EPI/SDOC + rats presented an opposite 5-HT innervation pattern within the NTS. Since the impairment of respiratory function seems to be moderate in EPI/SDOC− rats, in contrast to EPI/SDOC + rats, it is conceivable that the increase in 5-HT innervation in both the NTS and VLM may be part of the protective mechanisms of the respiratory function. However, EPI/SDOC + rats presented an increase in brainstem levels of both TPH2 and SERT transcripts. In EPI/SDOC + rats, such a mechanism may support an increase in 5-HT concentration in neuron terminals by increasing 5-HT synthesis and re-uptake of intact 5-HT molecules, and thus may represent an adaptive mechanism to partially compensate for the decreased brainstem 5-HT innervation. This conclusion is supported by results reported in conditional knock-out Lmx1b mice and WAR rats, which present respiratory alterations at rest and a concomitant decrease in 5-HT drive^31,47^, similar to that seen in EPI/SDOC + rats. We also believe that according to the difference in 5-HT innervation between VLM and NTS observed in the same EPI/SDOC + rats, the decrease in 5-HT could be structure-specific and requires further investigation in other primary regions of respiratory regulation.

We also compared the brainstem 5-HT system of DBA/2 mice with FRA to relevant control mice—DBA/2 mice with NFRA, in contrast to previous studies that compared DBA/2 mice with FRA to C57BL/6 mice that did not exhibit AGS^16^. We found an increased level of transcripts for TPH2 and SERT in DBA/2 mice with FRA. Surprisingly, 5-HT profile of EPI/SDOC + rats was similar to that of DBA/2 mice with FRA. Accordingly, alterations in the 5-HT system are likely involved in respiratory dysfunction in both acute and chronic seizure models and more likely associated to more severe respiratory phenotype in both strains.

In conclusion, the Pilo-SE rat model, which shows both chronic respiratory alterations and spontaneous seizures, could be a relevant model to further explore the neurobiological mechanisms underlying chronic alterations in respiratory regulation in epilepsy. Identifying the mechanisms that protect EPI/SDOC + rats from mortality may also be an interesting approach for new therapeutic strategies to prevent SUDEP.

## Methods

Protocols and methods in this study were performed in compliance with relevant institutional and ARRIVE guidelines and regulations. All experiments have been approved by the ethical committee of the Claude Bernard Lyon 1 University (protocol # BH-2008-11) and done in accordance with the European Communities Council for animal care (directive 2010-63), according to the French law (decree 2013-118).

### Animals

Five-week-old male Sprague–Dawley rats (Envigo, The Netherlands) were delivered to the animal facility of the Lyon 1 University (Villeurbanne, France) and housed in groups of five in transparent polycarbonate cages. Male DBA/2 mice of breeding colony of the LTSI laboratory were housed in groups of five. Rats and mice were maintained under a controlled 12-h/12-h light–dark cycle in a temperature-controlled room (24–26 °C and 21–23 °C for rats and mice, respectively), with free access to food and water.

### Experimental design

A full description of methods has been described in detail elsewhere^17,33,48–50^. Rats subjected to Pilo-SE were observed daily for spontaneous seizures, until development of epilepsy (EPI rats). The respiratory thermochemistry setup was used to measure oxygen consumption (⩒O_2_) during 24 consecutive hours (daily ⩒O_2_) for each rat. Animals in this study were subjected to one of the following experiments (See supplementary Fig. S4 online):

#### Experiment 1

To establish the time course of respiratory alterations in Pilo-SE rats, 4 series with 10 rats each were used in this experiment. In each series, Pilo-SE was induced in eight rats, and two rats were used as controls. For each series, the daily ⩒O_2_ for each rat was measured once a week from the 1^st^ to the 8^th^ week post-SE.

#### Experiment 2

To further investigate cardiorespiratory functions in EPI rats during the chronic phase of epilepsy, Pilo-SE was triggered in 30 rats, while 16 rats were used as controls. During the chronic period of epilepsy (12 weeks post-SE), resting ventilation (normoxia, 21% O_2_) was measured using whole-body plethysmography. Between 13–14 weeks post-SE, a subset of EPI and control rats were used to evaluate ventilatory responses to hypoxia (HVR, FiO_2_ = 9%) and hypercapnia (HCVR, FiCO_2_ = 5%), while the others underwent implantation of a telemetric device to evaluate cardiac frequency and body temperature at 15 weeks post-SE. As ⩒O_2_ and ventilation are intermingled processes, to visualize the global state of the respiratory system in EPI rats, we performed an in-depth analysis of our data by characterizing ventilatory patterns according to ⩒O_2_ patterns. Notably, daily ⩒O_2_ in EPI and control rats was measured shortly before plethysmography at 7–9 weeks post-SE.

#### Experiment 3

To determine whether the integrity of the brainstem 5-HT system is altered in EPI rats with respiratory alterations, Pilo-SE was induced in 20 rats, while eight rats were used as controls. Daily ⩒O_2_ was evaluated between the 6^th^ and 8^th^ weeks post-SE. Subsequently, animals were transcardially perfused with paraformaldehyde or NaCl and their brains were collected and processed for histology (5-HT immunodetection; n = 3 in each group), or for gene expression analysis (SERT and TPH2 mRNA; n = 5 in each group), respectively.

#### Experiment 4

To determine whether the integrity of the brainstem 5-HT system is compromised in DBA/2 mice with FRA, 11 DBA/2 mice underwent AGS followed by respiratory arrest. Brains from both FRA and NFRA DBA/2 mice were removed immediately after AGS. As for NFRA mice, mice with FRA were rescued and were injected with a lethal dose of pentobarbital and then perfused with NaCl. Brainstem SERT and TPH2 gene expression was measured and compared between mice with FRA and NFRA.

### In vivo procedures

#### Pilocarpine-induced SE

Epilepsy was triggered following Pilo-SE in 180–200 g male rats at the age of 7 weeks, as previously described^33,49^. Scopolamine methylnitrate (1 mg × kg^−1^, s.c.; S-2250, Sigma-Aldrich) was administered 30 min prior to pilocarpine hydrochloride (350 mg × kg^−1^, i.p.; P-6503, Sigma-Aldrich). After an initial period of immobility, the onset of SE was characterized by repetitive tonic–clonic activity of the trunk and the limbs, occurring after repeated rearing with forelimb clonus and falling. Because SE event is a life-threating condition in rats, there is a need to stop it after 2 h by a single injection of diazepam (Valium®, 10 mg × kg^−1^, i.p.; Roche). Rats were then hydrated with 2 mL of saline solution (0.9% NaCl; s.c.) and transferred to individual cages. After a one-week recovery period, all rats (control and treated rats) were housed in groups of 5 to avoid any stress that may result from social isolation.

#### Animal care after Pilo-SE

Control and treated rats were weighted daily during the first two weeks following Pilo-SE, and then every week until termination of the experiment. Daily abdominal massage was performed twice a day during the first week to activate intestinal motility, which is disrupted following Pilo-SE.

#### Detection of spontaneous recurrent seizures after Pilo-SE

To maintain as low as possible the level of anxiety in rats subjected to Pilo-SE, electro-encephalographic recordings (EEG) were not performed to detect the onset of epilepsy in our experiments. Indeed, conventional EEG set-up requires that rats are housed individually in a restrictive environment, which is known to enhance stress and anxiety and worsens epilepsy phenotype in Pilo-SE models^51^. In addition, as mentioned below in the “whole-body plethysmography” section, it is difficult to perform ventilatory recordings in animals with high anxiety level.

It is now clearly established that rats of the Sprague–Dawley strain from the Janvier Laboratories develop spontaneous recurrent seizures between 10 and 36 days after induction of Pilo-SE at 42 days of age^52,53^. Because we were using Sprague–Dawley rats from Envigo Laboratories, we had to monitor the time of onset of spontaneous recurrent seizures (SRSs) in our experiments. To determine whether rats were in the chronic phase of epilepsy, they were manipulated daily between 09:00 and 11:00 a.m. to define the onset of handling-induced seizures (HIS), which was induced by restraining rats at the level of the chest with gentle pressure for 10 s^9,52^. HIS were used in our study to help experimenters to define the time to start detecting the first behavioural spontaneous recurrent seizures (SRSs). Thus, upon detection of 2 HIS, rats were observed by experimenters for 5 consecutive hours (between 01:00 and 06:00 p.m.) over the following days to detect the presence or absence of behavioural SRSs. Following Pilo-SE, rats are considered epileptic once they developed at least two SRSs during the periods of the daily observations. The severity of these seizures was scored from 3 to 5 on the Racine's scale^54^. Seizure severity of less than 3 was difficult to detect visually in this study, which was conducted without daily video monitoring. Stage 3 corresponds to forelimb clonus, stage 4 to bilateral forelimb clonus with rearing, and stage 5 to rearing and falling or to tonic–clonic seizures.

During the 24 h-⩒O2 recordings, rats were video-monitored for 12 h during the light period, making it possible to evaluate the number of seizures and their severity per session.

### Evaluation of respiratory and cardiac functions

#### Respiratory thermochemistry

To detect respiratory alterations in epileptic rats, we used respiratory thermochemistry as an alternative approach to plethysmography, allowing respiratory function to be recorded for a long period of time on fed, unrestrained, and unanesthetized animal. Respiratory thermochemistry mostly reflects gas exchanges and allows more specifically the measurement of the oxygen consumption (⩒O_2_) with no bias effect from animal’s body movements on the accuracy of respiratory acquisition.

Oxygen consumption (⩒O_2_) was measured with sample rate of 3 acquisitions per minute, under standard conditions (variation in temperature = 0 °C; Ambient pressure = 760 mm Hg; dry air) using a thermochemistry open system, as previously described^55^. ⩒O_2_ was continuously recorded during 24 consecutive hours on the same thermochemistry system; therefore, only one rat could be recorded each day in our experimental setting. Briefly, rats were placed individually in a thermostatic recording chamber (12 L) with steady airflow (4 L × min^−1^), under a controlled 12-h/12-h light–dark cycle (light from 06:00 a.m. to 06:00 p.m.), and were allowed free access to food and water. Ambient temperature inside the thermostatic chamber was monitored with copper-constantan thermocouples and was maintained at 24–26 °C using a thermostatic container. Airflow rate was measured using a Platon volumeter and converted to standard STPD (standard temperature and pressure, dry) values. Fractional O_2_ concentration was measured using a Servomex 1100 paramagnetic gas analyzer (Taylor Instrument Analytics ltd, Sussex, UK) and ⩒O_2_ was calculated. The O_2_ analyzer was calibrated daily with pure nitrogen gas (0% O_2_) and room air (20.93% O_2_). Rats were weighted before and after recording then ⩒O_2_ values were normalized to the average body weight (calculated from the weight measured before and after each recording session). Rats were video-monitored during the daylight period.

#### Whole-body plethysmography

Plethysmographic chamber allows collecting ventilatory variables from acquired breathing pattern signal. Ventilatory function was evaluated between 09:00 a.m. and 04:00 p.m. in unrestrained, unanesthetized rats under BTPS (body temperature and pressure, saturated) conditions using whole-body plethysmography (EMKA; technology). Calibration of the system was performed before each recording session by injecting 1 mL air pulse inside the recording chamber. Briefly, each rat was individually placed in the recording chamber (600 mL) that is connected to a constant bias flow supply providing continuous inflow of fresh air (2.5 L × min^−1^). Minute volume (⩒_E_; mL × min^−1^) was calculated as the product of tidal volume (V_T_; mL) and respiratory frequency (f_R_; breath x min^−1^). Total respiratory cycle duration (T_tot_) was calculated as the sum of the inspiratory time (T_I_; msec) and expiratory time (T_E_; msec). We also calculated for each animal the coefficient of variation of the respiratory frequency, which is an index of the variability of the respiratory pattern^56^ and the frequency of spontaneous apnea, which is defined as a cessation of breathing lasting more than two respiratory cycles (> 2 × T_tot_). Apnea number and duration were calculated. Apnea frequency was normalized to recording duration and expressed per min of breathing. Recordings were started after a 40-min habituation period. Then, resting ventilation was monitored during 20 min under normoxic condition (21% O_2_). After recordings, rats were brought back to their home cage.

Ventilatory responses to hypoxia (FiO_2_ = 9%) and to hypercapnia (FiCO_2_ = 5%) were evaluated during two distinct recording sessions, which were performed a week apart from each other. After a 40-min habituation period, plethysmographic recordings under basal condition (normoxia) were started 10 min before gas variations. Ventilatory responses to a 10-min exposure to hypoxia or hypercapnia were subsequently evaluated. All plethysmography recordings were made between 09:00 a.m. and 04:00 p.m.; because, beyond 04:00 p.m., EPI rats were more stressed in the plethysmography chamber, requiring longer time to reach a quiet state in this novel and restricted environment. At the end of each recording session, rats were brought back to their home cage. Repeated measurements are randomly performed for each rat.

#### Abdominal implantation of telemetry transmitters to evaluate cardiac function and body-temperature

Rats were anesthetized with a mix of ketamine and xylazine (75 mg × kg^−1^ and 25 mg × kg^−1^, respectively; i.p.). A midline abdominal incision was made and a sterile telemetry transmitter (Data Sciences International, France) was placed inside the abdominal cavity. A pair of biocompatible wire electrodes originating from the transmitter were tunnelled subcutaneously and implanted to the left intercostal muscles and between the right shoulder blades. The transmitter was attached to the abdominal wall during wound closure with absorbable sutures. After surgery, animals received an intramuscular injection of penicillin (15,000 units) once a day for 3 consecutive days and were then returned to their home cage. Rats were monitored daily to control general health state. Two weeks after surgery, telemetry transmitters were magnetically activated and freely moving animals were placed in individual cages placed under platform-receiver units. Variation in cardiac frequency and body-temperature were recorded during 24 consecutive hours.

### Induction of audiogenic seizures in DBA/2 mice

AGS were induced as previously described^17,22^, with slight modifications. Briefly, at age of 23 days, mice were individually placed in a plastic cylinder (40 cm diameter, 20 cm height) and exposed to a broadband acoustic stimulus (intensity of 110 dB SPL with a peak frequency at 12 kHz) generated by a mechanical sonicator (Deltasonic model Delta 920). The stimulus was given for a maximum duration of 60 s or until the mouse exhibited AGS, characterized by an initial wild running and ending by a tonic hindlimb extension. All mice exhibited a tonic hindlimb extension leading to a respiratory arrest identified by pinnae relaxation and a complete cessation of chest movements. Respiratory arrest was followed by spontaneous recovery of the respiratory function within 5 s in mice identified as NFRA, or was followed by death for the other mice (FRA). Mouse brain was systematically removed within 2–3 min after acoustic stimulus was triggered.

### Ex vivo procedures

DBA/2 mice and rats were injected with a lethal dose of pentobarbital (250 mg × kg^−1^; i.p.) before being sacrificed. For gene expression analyses, animals were transcardially perfused (30 mL min^−1^) for 2 min with ice-cold NaCl and their brain was rapidly removed. Then, the brainstem region was isolated, frozen in liquid nitrogen and stored at − 80 °C. For immunohistochemistry procedure, animals were transcardially perfused (30 mL min^−1^) for 2 min with ice-cold NaCl and then for 10 min with ice-cold 4% paraformaldehyde in 0.2 M phosphate buffer (30 mL min^−1^). After cryoprotection in a 25% sucrose solution, brains were frozen at − 40 °C in isopentane and stored at − 80 °C.

#### 5-HT immunohistochemistry

Coronal sections (40 µm thick) were cryostat-cut throughout the medulla oblongata and were processed free-floating for 5-HT immunostaining. Using the rat brain atlas, sections from each experimental group were selected at interaural coordinates − 2.60 mm, and were all processed together for 5-HT immunofluorescent labeling. Selected sections were rinsed in 0.1 M Phosphate Buffered Saline (PBS) and incubated in PBS containing 0.3% Triton X-100 and 3% normal donkey serum. Then, sections were incubated overnight at 4 °C with a rabbit polyclonal antibody against 5-HT (Sigma; S5545; final dilution = 1:3000). After few washes, sections were incubated overnight at 4 °C with an AlexaFluor-488 conjugated donkey anti-rabbit antibody (Molecular Probes; A-21206; final dilution = 1:1000). At the end of the immunostaining protocol, sections were mounted onto SuperFrost®Plus slides and cover slipped using Vectashield mounting medium (H-1500, Vector).

#### Semi-quantification of brainstem 5-HT immunostaining

Images (1152 × 1024 pixels) were captured with a HLX PL APO × 63/0.70 objective and with the same conditions of photomultiplier gain, offset and pinhole aperture using a TCS SP2 confocal microscopy system (Leica), allowing the comparison of fluorescence intensity between sections. Image analysis was conducted on 8 z-compressed images stacks (each image = 4 µm depth), which include the whole region of interest. Using the image J software (NIH), we first measured the average grey value within each 5-HT-positive neuronal cell body of caudal raphe nuclei (pallidus, magnus and obscurus), as an index of 5-HT neuronal concentration (A.U.). Using the same software, we also measured the density of 5-HT-immunopositive processes within the surface area of the NTS and the VLM, expressed as 5-HT-positive pixel/ pixels^2^.

#### Quantification of transcript level variation by RT-qPCR

Total RNA was extracted from brainstem samples using Tri-reagent LS (Molecular Research Center, Inc.). Contaminant genomic DNA was subsequently removed from the samples by treatment with Turbo DNA-*free*™ kit (Ambion). The messenger RNAs (mRNAs) contained in 1 µg of purified RNA extracts were reverse-transcribed using the reverse transcriptase RNase H minus (Promega) and oligo-d(T)15. To normalize the RT step, a synthetic external and non-homologous poly(A) standard RNA (SmRNA; Morales and Bezin, patent WO2004.092414) was added to the RT reaction mix (150,000 copies in each experimental sample), as previously described^33,48–50^. PCR amplification of selected cDNAs, i.e. those complementary to the mRNA encoding rate-limiting enzyme of brainstem 5-HT synthesis (tryptophan hydroxylase: TPH2) and the 5-HT transporter (SERT), were performed using the Rotor-Gene Q system (Qiagen) and the QuantiTect SYBR Green PCR Kit (Qiagen). Sequences of the different primer pairs used for PCR amplification of mouse and rat TPH2 and SERT cDNAs are listed in Supplementary Table S1 online.

### Statistical analysis

The data for each experimental group were pooled and expressed as the mean ± SEM in the text and figures, unless otherwise specified. All statistics were performed using Sigma plot software 3.5 (Systat Software Inc., Point Richmond, California, USA). A Kolmogorov–Smirnov test was used to test if the data follows a normal distribution, then paired or unpaired t-test was performed for the two-group comparison. For multiple group comparisons, one-way ANOVA followed by post-hoc Fisher’s LSD test was used. Statistical significance was predefined as P < 0.05.

## Supplementary Information

Supplementary Information.

## Data Availability

The data that support the findings of this study are available from the corresponding author upon reasonable request.

## Abbreviations

5-HT: Serotonin
AGS: Audiogenic seizures
EPI: Epileptic
CIH: Chronic intermittent hypoxia
EEG: Electro-encephalography
EPI/SDOC−: Epileptic rat without sharp decrease in oxygen consumption
EPI/SDOC+: Epileptic rat with sharp decrease in oxygen consumption
f_R_: Respiratory frequency
FRA: Fatal respiratory arrest
HCVR: Hypercapnia ventilatory response
HIS: Handling-induced seizures
HVR: Hypoxia ventilatory response
NFRA: Non-fatal respiratory arrest
GTCS: Generalized tonic–clonic seizures
LTSI: Laboratoire de Traitement du Signal et de l ‘Image
NTS: Nucleus tractus solitarius
OSA: Obstructive sleep apnea syndrome
Pilo-SE: Pilocarpine-induced *status epilepticus*
SE: *status epilepticus*
SERT: Serotonin transporter
SSRI: Selective serotonin reuptake inhibitor
SUDEP: Sudden unexpected death in epilepsy
T_E_: Expiration time
T_I_: Inspiration time
TLE: Temporal lobe epilepsy
Tph2: Tryptophan hydroxylase 2
T_tot_: Total respiratory cycle duration
⩒_E_: Minute volume
VLM: Ventrolateral medulla
⩒O_2_: Oxygen consumption
V_T_: Tidal volume
