# Supplementary material for: Respiratory dysfunction in two rodent models of chronic epilepsy and acute seizures and its link with the brainstem serotonin system
Source: Sci Rep. 2022 Jun 17;12:10248. doi: 10.1038/s41598-022-14153-6 (PMC9205882; doi:10.1038/s41598-022-14153-6)
Supplement: Supplementary file 1 — Supplementary Information. [file 41598_2022_14153_MOESM1_ESM.docx]

**Supplementary information**

**Respiratory dysfunction in two rodent models of chronic epilepsy and acute seizures and its link with the brainstem serotonin system**

Hayet Kouchi ^1,2^, Michaël Ogier ^3^, Gabriel Dieuset ^4^, Anne Morales ^1,2^,
Béatrice Georges ^1,2^, Jean-Louis Rouanet ^5^, Benoît Martin ^4^, Philippe Ryvlin ^6^,
Sylvain Rheims ^1,2,7#^, Laurent Bezin ^1,2#*^

1. Lyon Neuroscience Research Center, Inserm U1028, CNRS UMR5292, Lyon 1 University, Lyon, France ;
2. Epilepsy institute, Bron, France;
3. IRBA, Institut de Recherche Biomédicale des Armées, Brétigny-sur-Orge, France;
4. Univ Rennes, CHU Rennes, Inserm, LTSI (Laboratoire de Traitement du Signal et de l‘Image), UMR-1099, Rennes, France;
5. Laboratoire d'Ecologie des Hydrosystèmes Naturels et Anthropisés, U5023, Lyon 1 University, Lyon, France;
6. Department of Clinical Neurosciences, Centre Hospitalier Universitaire Vaudois, Université de Lausanne, Lausanne, Switzerland.
7. Department of Functional Neurology and Epileptology, Hospices Civils de Lyon, Lyon, France.

**^#^** co-last authors

*Corresponding author: Laurent Bezin and Hayet Kouchi

**Supplementary results**

**Development of behavioral spontaneous recurrent seizures in rats after pilocarpine-induced *status epilepticus***

Briefly, in experiment 1, 32 rats were subjected to pilocarpine-induced *status epilepticus* (Pilo-SE); eight of them did not develop SE and four died during SE. By the end of the 2^nd^ week post-SE, the 20 remaining rats developed spontaneous recurrent seizures (SRSs), indicating that they became epileptic (EPI). In experiment 2, 30 rats were subjected to Pilo-SE; 7 were excluded either because they did not develop SE (5/7) or because they died during SE (2/7). The remaining rats developed epilepsy (n=23 EPI rats) by the end of the 2^nd^ week post-SE. In experiment 3, 20 rats were subjected to Pilo-SE; 2 of them did not develop SE and 2 others died during SE. The remaining rats (n=16) developed epilepsy by the end of the 2^nd^ week post-SE.

**Non-ventilatory variables related to** ⩒**O_2_**

In the 3 groups of rats (controls, EPI/SDOC+ and EPI/SDOC-), we evaluated the effect of three factors that have been referenced as affecting ⩒O_2_ ^1-7^ : time (t_0_ corresponds to the time of induction of SE in EPI rats), body weight and circadian rhythm. We also evaluated the effect of seizures on ⩒O_2_.

***Time effect.*** It has been described in the main text. Time had no effect both on the frequency and the duration of SDOCs.

***Body-weight effect.*** Because ⩒O_2_ of each rat is systematically reported to the body-weight, we analyzed body-weight variations in all groups of rats throughout the course of the experiments. Maximum body-weight loss in rats was observed three days after induction of SE, reaching -23 ± 2% of their initial weight (measured the day before SE). They then regained weight to return to control values from 49 days post-SE. It is to note that retrospective analysis of EPI rats showed no significant difference in body-weight variation between EPI/SDOC+ and EPI/DSOC- rats (p=0.93), indicating that the decrease in ⩒O_2_ in EPI/SDOC+ rats cannot be related to higher weight gain compared to EPI/SDOC-.

***Circadian rhythm effect.*** Since the daily ⩒O_2_ pattern is known to be under the control of circadian rhythm ^4-7^, we compared ⩒O_2_ between the light and the dark periods for each group of rats. At 1-2 weeks post-SE, the average ⩒O_2_ increased during the dark period compared to the light period in all groups of rats (control rats: +21 ± 7 %, p<0.001; EPI/SDOC- rats: +28 ± 2 %, p<0.001; EPI/SDOC+ rats: +24 ± 7 %, p=0.01). At 5-8 weeks post-SE, such differences between the dark and the lights periods were still observed for the three groups of rats (control rats: +21 ± 2 %, p=0.001; EPI/SDOC- rats: +23 ± 3 %, p<0.001; EPI/SDOC+ rats: +17 ± 5 %, p=0.006). The regulation of circadian rhythm was statistically similar between groups. Regarding SDOCs occurrence, the severity of these events was not different between light and dark periods in EPI/SDOC+ rats.

***Seizure effect.*** To characterize the ⩒O2 pattern during seizure manifestation in EPI rats, we based our analysis on the concomitant 12h-daylight video-thermochemistry sessions. A total of 10 seizures occurred under video monitoring. These seizures have been exhibited by a total of 8 EPI rats (5 EPI/SDOC-, 3 EPI/SDOC+ rats). Similar to our previous visual observations during the first two weeks following SE, no significant difference was noticed for the number of seizures between EPI/SDOC+ and EPI/SDOC- rats (p=0.52). Both EPI/SDOC+ and EPI/SDOC- rats exhibited stage 4-5 seizure severity according to Racine’s scale. The average duration of seizures was 77 ± 0.08 sec with no difference between EPI/SDOC+ and EPI/SDOC- rats (p=0.67). To determine ⩒O_2_ variation during seizure in EPI/SDOC- and EPI/SDOC+ rats, the segments of ⩒O_2_ before, during and after seizures were isolated for characterization. Short after seizure, ⩒O_2_ increased to reach a plateau whose values were +61 ± 7 % higher than those measured during the 10 min prior to seizure occurrence. This increase was particularly sustained across time, with an average duration of 37 ± 5 min before returning to basal values. There was no correlation between seizure severity and the extent or duration of ⩒O_2_ increase.

**References**

1 Feher, J. J. *Quantitative human physiology : an introduction*. Second edition. edn, (Elsevier/AP, Academic Press is an imprint of Elsevier, 2017).

2 Gastinger, S., Sorel, A., Nicolas, G., Gratas-Delamarche, A. & Prioux, J. A comparison between ventilation and heart rate as indicator of oxygen uptake during different intensities of exercise. *J Sports Sci Med* **9**, 110-118 (2010).

3 Gautier, H. Interactions among metabolic rate, hypoxia, and control of breathing. *Journal of applied physiology* **81**, 521-527, doi:10.1152/jappl.1996.81.2.521 (1996).

4 Refinetti, R. Metabolic heat production, heat loss and the circadian rhythm of body temperature in the rat. *Experimental physiology* **88**, 423-429, doi:10.1113/eph8802521 (2003).

5 Refinetti, R. The circadian rhythm of body temperature. *Front Biosci (Landmark Ed)* **15**, 564-594, doi:10.2741/3634 (2010).

6 Seifert, E. L. & Mortola, J. P. Circadian pattern of ventilation during acute and chronic hypercapnia in conscious adult rats. *Am J Physiol Regul Integr Comp Physiol* **282**, R244-251, doi:10.1152/ajpregu.00290.2001 (2002).

7 Stephenson, R., Liao, K. S., Hamrahi, H. & Horner, R. L. Circadian rhythms and sleep have additive effects on respiration in the rat. *The Journal of physiology* **536**, 225-235, doi:10.1111/j.1469-7793.2001.00225.x (2001).

**Supplementary figures and table**

**Figure S1:** **Metabolic rate of oxygen at 1–2 weeks and 5–8 weeks post-SE.** Average oxygen consumption (⩒O2) was calculated for the light and dark periods at 5**–**8 weeks post-SE (A) and then at 1**–**2 weeks post-SE (B) using the same experimental groups, retrospectively. Dichotomization of SDOC+ and SDOC- EPI rats was based on data collected at 5**–**8 weeks post-SE, which showed a significant decline in the average ⩒O2 of EPI/SDOC+ rats during both the light and dark periods, compared to EPI/SDOC- and control rats (A). There was no difference between the three rat groups at 1**–**2 weeks post-SE, when no SDOCs were detected (B). Results are expressed in percent of control rats *, P<0.05; ***, P<0.001, compared to control rats. ✝✝✝, P<0.001, compared to EPI/SDOC- rats.

**Figure S2: Ventilatory function in epileptic and control rats under normoxia**. (A) Ventilation (V_E_), respiratory frequency (F_R_) and tidal volume (V_T_) in epileptic (n=16) and control (n=5) rats at 12 weeks post-SE*.* (B) Index of pattern stability includes coefficient of variation of ventilatory pattern and apnea number/duration. Results are expressed as mean ± SEM. *, P<0.05; **, P<0.01, in comparison to control rats.

**Figure S3: 5-HT immunopositive cells in raphe nuclei within the medulla oblongata in control and EPI rats.** Fluorescent 5-HT labeling was performed in sections selected at interaural -2.60 mm within caudal raphe nuclei. Images of 5-HT positive cells were captured using confocal microscopy (x63 objective).

**Figure S4: Experimental design using rats.**

**Supplementary Table S1: Primer pairs used for qPCR amplification of rat and mouse TPH2 and SERT cDNAs.**
